# Progress in Surgery

**Published:** 1894-02-17

**Authors:** 


### Progress in Surgery.

Injuries to the Epiphyses and their Results Are reviewed by Mr. Jonathan Hutchinson, jun.,[19] in his "Lectures at the Royal College of Surgeons." He bases his remarks upon the collected records of about 350 cases, and alludes to a number of experiments he made upon the cadaver and in living animals, which help to clear up disputed and doubtful facts, not only in the mechanism and anatomy of these injuries, but also in the function of the epiphysial cartilage, or, as he prefers to term it, the epiphysial disc. Although many cases and specimens show no impairment of the disc, yet arrest of growth is a fairly frequent result of detachment of the epiphysis. It is almost inevitable if the latter remains displaced, but it may sometimes occur even when there has been no decided displacement, or when this has been completely overcome. Reduction should be effected as soon as possible, for the following reasons: (1) The rapid occurrence of swelling will make the diagnosis and correction of the exact displacement more difficult; (2) the connecting bridge of periosteum quickly thickens and shortens; (3) the more prompt the replacement the less will be the risk of exuberant callus, of interference with growth, and possibly also of suppuration and necrosis. The more important any one epiphysial disc is as regards the growth of the bone the greater is the risk of suppuration following in non-compound cases. He adds one case of separation of the epiphysis of the clavicle to those already published. The chief points in the diagnosis of separation of the upper epiphysis of the humerus, from an analysis of sixty-six cases are: (1) Age of patient, under about twenty years; (2) arm comparatively helpless; elbow often directed a little outwards and backwards; (3) abnormal mobility just below shoulder-joint on abduction; (4) rapid swelling about shoulder and shortening if diaphysis is wholly displaced; (5) muffled crepitus. The necessity for thorough examination, correct diagnosis, and gentleness in manipulation in every case of detachment of this epiphysis is emphasised. After reduction by traction, &c., a well-fitting poroplastic shoulder-cap should be applied. The nature of the epiphysial junction will tend to keep the diaphysis in place. With regard to irreducible, compound, complicated cases, and certain cases of long standing in young subjects with impaired movements, operative measures are advised. Clean separation of the lower epiphysis of the humerus exactly at the epiphysial disc is uncommon after the age of five or six; in fifty cases it was present probably only in the minority. The youngest patient was eighteen months old, the oldest fourteen years. Shortening of the arm is of no importance in practice, as the lower epiphysial line of the humerus may be disregarded after the tenth year. In the great majority there is more or less complete displacement backwards of the epiphysis, but not infrequently some lateral displacement either outwards or inwards, and very infrequently the epiphysis is driven forwards. The symptoms of this injury resemble and are frequently mistaken for dislocation. The complications, recent and remote, together with the prognosis are discussed by Mr. Hutchinson. He believes in a well-moulded splint to the back of the forearm and arm in the flexed position, or a back splint with an interruption at the elbow, and condemns the treatment by full extension. In six out of fourteen of his own cases, the results as regards absence of deformity and the elbow movements, were perfect, in eight extension and flexion were only slightly limited. The age of thirty-eight cases of detachment of the internal epicondyle, whose records Mr. Hutchinson has collected, ranged between eight and eighteen years. The elbow-joint is probably always opened, and union by bone takes place only in rare cases. The frequency of dislocation of the forearm and injury to the ulnar nerve are most important points; for the latter complication he excised the epicondyle in two cases with good result. Detachment of the anterior inferior spinous process of the ilium is probably not so rare as it has been hitherto believed. (R. H. A.) Whitelocke[20] relates two cases caused by violent muscular action, in patients aged respectively eighteen and nineteen years. Crepitus, mobility of a small piece of bone, and discolouration, with swelling below the outer side of Poupart's ligament, and in one case numbness and tingling down the front of the thigh, were the chief signs. The erroneous if not disastrous view of regarding separation of the lower epiphysis of the femur, with displacement, as equivalent to fracture near the joint, is commented upon by Mayo Robson.[21] In addition to several published examples he reports three cases, in two of which the displacement of the epiphysis was forward, in the third this was incomplete. He had seen three other cases, in all of which there was the same displacement. Pressure upon the poplitæal vessels by the upper fragment and arrest of circulation in the leg existed in the first case. Reduction was effected under ether, and the leg suspended in a Macintyre's splint for five weeks. Four months later no deformity or shortening existed. In the second and third cases reduction was also successful, and a useful limb resulted. Robson advocates first reduction under an anæsthetic, and then the use of the long splint with weight and pulley or the double inclined plane. The tendo Achilles may be divided. Should reduction be impossible then excision might be adopted; if the large vessels are ruptured, or gangrene occur, amputation can be the only resource. An interesting case of rotatory dislocation between the atlas and axis is reported by Cyrus Legg[22]; it appears to have been caused by a violent twist of the head. The head remained twisted to the left side, and every attempt to rotate caused great pain; the chin was slightly raised and the sterno-mastoid and trapezius on the right side quite tense. Reduction was effected by vigorous extension and a jerking movement, while counter extension was made at the shoulders. Instant relief was obtained, a retention bandage applied, and the lad did well.

### Joints.

Erasion of joints is rapidly replacing excision. At a recent meeting of the Royal Medical Chirurgical Society H. H. Clutton[23] introduced the subject of arthrectomy of the elbow and ankle joints, and advocated the former as an early operation in place of a late excision. Out of nine cases of arthrectomy of the elbow excellent results were obtained in six; in two of the others the joint was subsequently excised. If the cartilages were not diseased a movable joint might be expected. Passive movement was not desirable if the wound followed an aseptic course. Six cases of disease of the ankle joint were submitted to arthrectomy, and the use of a knee-rest for a year after the operation was considered necessary. Although the tendency nowadays is to regard tubercle of bone as the usual starting point of most tubercular joints, yet in the knee and some other joints, as Mr. Barling says,[24] the synovial membrane is more frequently the starting point. In the hip, excision is necessary, if an attempt is made to eradicate the disease, a complete arthrectomy being impossible; whilst in the knee-joint complete arthrectomy is not only possible but is more efficient than excision as usually performed. Two valuable papers have recently been published on the treatment of hip disease. The first, by E. H. Bradford,[25] on traction in hip disease, gives a *résumé* of the experiments of König, Lannelongue, Brackett, and others, showing the efficacy of a continuous traction of 10 to 20 lbs., not only in overcoming muscular spasm, but also in causing separation of the articular surfaces. If the limb is slightly abducted and slightly flexed, traction distracts to a noticeable degree, but if the limit is extended to its utmost limit no amount of force will distract. From experimental and clinical evidence he concludes that in the active stage of hip disease, as well as in disease of a severe type attended by suppuration, properly applied traction should be employed. Deformity resulting from dislocation may be prevented if traction be carried out with care for years. Taylor[26] also insists upon mechanical counter extension and fixation being applied in ostitis of the knee with precision in the lines of present deformity, or so near this as to be perfectly comfortable. In the second paper by (A. B.) Judson,[27] three cases are quoted, illustrating the good effects of mechanical treatment, even in the most unpromising cases of hip disease. In advanced conditions symmetry of the limb and power of natural locomotion will often result. The patient should not be confined to bed. Fixation of the joint, or a reasonable degree of immobilization, which is all that is required, is easily maintained by Judson's splint. This is essentially an ischiatic crutch, consisting of a straight steel upright (thickened antero-posteriorly), extending from the ground to midway between the iliac crest and trochanter, and a pelvic steel band supporting the perineal strap, upon which the patient sits during progression. His weight alternately rests on the sound foot and perineal strap. The upright is movable as an upper box piece, and lower bar adjusted by a rack and pinion. A table of 146 cases of tubercular disease of the hip gives, according to Sherman,[28] for excision in 64 cases, both as to movement and progression, a result decidedly in favour of operation. Two forms of sacro-iliac arthritis occurring after labour are distinguished by Budin[29]. The first is suppurative, and non-recoverable, being due to severe infection; the second is not so dangerous, and has most frequently been observed after difficult, especially forceps deliveries. Gonorrhœal sacro-iliac arthritis observed by Fournier is quite exceptional Puerperal sacro-iliac arthritis is chiefly characterised by pain increased on walking and standing, by direct pressure on the articular line either back or front, and by indirect pressure upon the lower limb or iliac crests. Treatment consists in absolute rest, immobility, and the use of mineral water baths. Bowlby's paper on syphilitic joint disease[30] has again drawn attention to an affection too little observed. The disease in Bowlby's patient was of congenital origin, and affected many joints. The lesions of the cartilage resembled, but were not quite identical with, the specimens described by Virchow. Clinically there had been no evidence of such extensive lesions as were found post mortem. He suggests that the lesion in the chronic arthritis of children with congenital syphilis is very probably of the same type. Sokoloff[31] records under the name of Fibroma arborescens two cases of localised and extensive papillomatous growths of the shoulder joint. Variation in intra-articular pressure, and not inflammatory irritation, is the cause of their development. The affection was observed in one case in a woman suffering from syringomyelia, and in the other in a man aged 65 with long-standing unreduced dislocation of the humerus. The polypoid masses occurred only in quite restricted localities springing from the synovial membrane where articular pressure did not exist, and in that part of the capsule which had ceased to functionate.

19 Brit. Med. Journ., July 8, 1893, and Dec. 30, 1893. 20 Brit. Med. Journ., Nov. 25, 1893, p. 1302. 21 Annals of Surgery, July, 1893, p. 1. 22 Lancet, Dec. 2, 1893, p. 1382. 23 Lancet, Dec. 16, 1893, p. 1510. 24 Birmghm. Med. Review, Oct. 1893, p. 217. 25 Amer. Med.-Surg. Bulletin, Nov., 1893, p. 1030. 26 New York Med. Journ., Nov., 1893, p. 597. 27 New York Med. Record, Oct. 28, 1893, p, 545. 28 Amer. Med.-Surg. Bulletin, Nov., 1893, p. 1097. 29 Amer. Med. and Surg. Bulletin, Sept., 1893. Progrès Medicale, April, 1893. 30 Lancet, Oct. 28, 1893, p. 105. 31 Annals of Surgery, Dec., 1893., p. 672; Sammlung Klin. Vorträge, No. 81, Sept., 1893.

